# Posttranscriptional Regulation and RNA Binding Proteins in Cancer Biology

**DOI:** 10.1155/2015/897821

**Published:** 2015-07-27

**Authors:** Claudia Ghigna, Luca Cartegni, Peter Jordan, Maria Paola Paronetto

**Affiliations:** ^1^Istituto di Genetica Molecolare, Consiglio Nazionale delle Ricerche (IGM-CNR), Via Abbiategrasso 207, 27100 Pavia, Italy; ^2^Department of Chemical Biology, Ernest Mario School of Pharmacy, Rutgers, The State University of New Jersey, Piscataway, NJ 08854, USA; ^3^Department of Human Genetics, National Health Institute Dr. Ricardo Jorge, 1649-016 Lisbon, Portugal; ^4^Laboratory of Cellular and Molecular Neurobiology, Santa Lucia Foundation, 00143 Rome, Italy; ^5^University of Rome “Foro Italico”, Piazza Lauro de Bosis 15, 00135 Rome, Italy

Following the completion of the human genome sequence and the concomitant technological innovations required for whole genome analyses, the last decade has witnessed an explosion of data and information concerning the posttranscriptional regulation of gene expression, in both pathological and nonpathological contexts. Among the most notable posttranscriptional events studied are the widespread usage of alternative splicing, the pleiotropic regulatory roles of miRNAs, and breakthroughs in the understanding of the control of gene expression by noncoding RNA transcripts.

In this special issue of this journal, the spotlight is centered on the role that various mechanisms of* posttranscriptional regulation*—and the* RNA binding proteins (RBPs)* that control them—play in* cancer biology*.

Adaptive changes in gene expression programs are crucial during tumor development, in order to allow cancer cells to support growth, survival, and metastasis and to resist to therapeutic treatments. Cancer cells have now been shown to efficiently adapt the expression of their proteome through changes in alternative splicing patterns, modifications of mRNA translational efficiency, or feedback modulators such as miRNAs and noncoding RNAs.

RBPs play a pivotal role in these processes and many of them are found aberrantly expressed in several tumor types. Moreover, each RBP most likely regulates a discrete but often broad subset of target transcripts at the same time, thus leading to an expanding functional network of changes that have important consequences for cancer cell biology.

Several of these changes occur at the level of alternative splicing, with the generation of variants that promote multiple aspects of tumorigenesis. V. Pagliarini et al. in “Splicing Regulation: A Molecular Device to Enhance Cancer Cell Adaptation” review some of the most striking and best-characterized examples of altered splicing events, which allow cancer cells to rapidly adapt to the adverse conditions encountered during the transformation process, leading to chemoresistance. In this regard, the authors discuss the possibility of new therapeutic protocols combining canonical chemotherapy with novel tools targeting this adaptive splicing response.

M. R. da Silva et al. in “Splicing Regulators and Their Roles in Cancer Biology and Therapy” explore the significance of cancer-associated alternative splicing events and then focus on the role of the major family of splicing regulators, SR proteins, and the kinases that regulate their activities. Their impact on cancer progression, as well as their possible use as targets for novel anticancer therapies, are discussed.

V. Gonçalves and P. Jordan in “Posttranscriptional Regulation of Splicing Factor SRSF1 and Its Role in Cancer Cell Biology” zoom in on the specific role played by one of the best-characterized SR-family proteins, SRSF1 (formerly SF2/ASF). Their review encompasses the posttranscriptional modifications and deregulated expression that contribute to transforming this essential splicing regulator into a powerful oncoprotein.

Our current understanding of how SAM68, a multifunctional member of the separate STAR (signal transduction and activation of RNA metabolism) family of RBPs, affects key cellular regulatory circuitries and promotes cancer development and progression is summarized by P. Frisone et al. in “SAM68: Signal Transduction and RNA Metabolism in Human Cancer.” In particular, the authors address how the transcriptional and posttranscriptional regulation of gene expression mastered by SAM68 contributes to changes occurring in cancer cells, thus opening the possibility of new therapeutic approaches targeting SAM68 activities in cancer.

Finally, the original research paper by E. Hong et al., “Unravelling the RNA-Binding Properties of SAFB Proteins in Breast Cancer Cells,” sheds light on the RNA-map of SAFB protein in breast cancer cells and highlights the contribution of this relative newcomer to the complex deregulated landscape of RNA processing in tumors.

Another set of manuscripts addresses regulatory events involved in mRNA translation.

M. J. Halaby et al. in “Translational Control Protein 80 Stimulates IRES-Mediated Translation of p53 mRNA in Response to DNA Damage” contribute an original research paper on the regulation of cap-independent p53 protein translation upon genotoxic stress. In particular, they identify two novel regulators of the p53 Internal Ribosome Entry Site (IRES): the translational control protein 80 (TCP80) and the RNA helicase A (RHA). The functional interaction between these two proteins becomes relevant for p53 induction and its tumor suppressive function in response to DNA damage.

In addition, F. Han et al. in “Emerging Roles of MicroRNAs in EGFR-Targeted Therapies for Lung Cancer” review our current knowledge concerning the role of the deregulation of the EGFR signaling pathway in lung cancer. In particular, they discuss the involvement of miRNAs in the development of drug resistance to anti-EGFR agents in lung cancer cells, indicating their possible application as predictive biomarkers for anti-EGFR therapy.

Finally, the original research paper by P. Cremaschi et al., “An Association Rule Mining Approach to Discover lncRNAs Expression Patterns in Cancer Datasets,” employs a bioinformatic approach (ARM (Association Rule Mining) methodology) for the meta-analysis of gene expression data. The ARM algorithm was applied for the study of differential expression profile of long noncoding RNAs (lncRNAs) in multiple tumor types and resulted in the identification of lncRNAs patterns differentially expressed in tumor versus normal tissues.

We hope that this special issue will contribute to a more thorough understanding of the role of posttranscriptional regulation and RBPs in tumorigenesis. In particular, a better comprehension of the molecular events that underlie malignant transformation will reveal potential novel drug targets for the development of more selective and effective anticancer therapies or identify novel biomarkers for disease progression or personalized patient stratification.

*Claudia Ghigna* *Luca Cartegni* *Peter Jordan* *Maria Paola Paronetto*

